# Lipase Catalyzed Transesterification of Model Long-Chain Molecules in Double-Shell Cellulose-Coated Oil-in-Water Emulsion Particles as Microbioreactors

**DOI:** 10.3390/ijms232012122

**Published:** 2022-10-12

**Authors:** Itzhak Meir, Gilad Alfassi, Yael Arazi, Dmitry M. Rein, Ayelet Fishman, Yachin Cohen

**Affiliations:** 1 Department of Chemical Engineering, Technion—Israel Institute of Technology, Haifa 3200003, Israel; 2 Department of Biotechnology Engineering, Braude College of Engineering, Karmiel 2161002, Israel; 3 Department of Biotechnology and Food Engineering, Technion—Israel Institute of Technology, Haifa 3200003, Israel

**Keywords:** lipase, cellulose, transesterification, emulsions

## Abstract

Lipase-catalyzed transesterification is prevalent in industrial production and is an effective alternative to chemical catalysis. However, due to lipases’ unique structure, the reaction requires a biphasic system, which suffers from a low reaction efficiency caused by a limited interfacial area. The use of emulsion particles was found to be an effective way to increase the surface area and activity. This research focuses on cellulose as a natural surfactant for oil-in-water emulsions and evaluates the ability of lipase, introduced into the emulsion’s aqueous phase, to integrate with the emulsion microparticles and catalyze the transesterification reaction of high molecular weight esters dissolved in the particles’ cores. Cellulose-coated emulsion particles’ morphology was investigated by light, fluorescence and cryogenic scanning electron microscopy, which reveal the complex emulsion structure. Lipase activity was evaluated by measuring the hydrolysis of emulsified *p*-nitrophenyl dodecanoate and by the transesterification of emulsified methyl laurate and oleyl alcohol dissolved in decane. Both experiments demonstrated that lipase introduced in the aqueous medium can penetrate the emulsion particles, localize at the inner oil core interface and perform effective catalysis. Furthermore, in this system, lipase successfully catalyzed a transesterification reaction rather than hydrolysis, despite the dominant presence of water.

## 1. Introduction

Lipase-catalyzed transesterification has become an effective alternative to chemical catalysis. It requires less energy, is less toxic, has higher selectivity and is the most prevalent biocatalyst in industrial production [1,2]. Although it is extensively investigated in the production of biodiesel, lipase transesterification is used to catalyze other novel materials, such as antibacterial food additives [3], natural antioxidants [4] and even wax esters, that are suitable for the cosmetics, pharmaceutics and lubricants industries [5]. The formation of these materials involves dissolving their substrates in organic solvents (since most of them are poorly water-soluble), which conflicts with enzymatic reactions that naturally require an aqueous environment. Therefore, the water content has a critical role in this system, as it is necessary for lipase stability and activity but promotes hydrolysis rather than transesterification if it exceeds a certain concentration [6]. Furthermore, the use of organic solvents is expensive, toxic, flammable and involves higher investment costs to meet safety requirements [7]. In industrial aspects, lipase transesterification has some major drawbacks regarding enzymes costs and the loss/inhibition of activity, as well as the used solvents that can be expensive, dangerous and flammable. Thus, enzyme recycling should be considered [8], as well as minimizing solvent usage [4,9]. Lipase (EC 3.1.1.3) has a strong hydrophobic region near the catalytic center, which has a characteristic of “interface activation” [2]. Therefore, the reaction often requires a biphasic system, which suffers from low reaction efficiency due to a limited interfacial area. To overcome this limitation, surfactants are used to increase the interfacial area by creating emulsions [10]. In such systems, lipase adsorbs onto the hydrophobic phase due to its amphiphilic character and is immobilized at the interface where it catalyzes the relevant reactions. Lipase activity in the effective hydrolysis of emulsified fish oil was reported [11]. Recently, the membrane emulsification process was shown to provide an emulsion microbioreactor in which lipase acted as both the catalyst and surfactant, exhibiting high (100%) enantioselectivity and conversion in the hydrolysis of (S)-naproxen methyl ester [12].

The surfactants used to fabricate and stabilize emulsions are sometimes toxic and may inhibit lipase activity. Inhibition was suggested to occur by various mechanisms, such as: the prevention of adsorption to the oil interface, formation of interfacial complexes or direct interaction and inactivation of the lipase’s active site [13]. To overcome the surfactants’ drawbacks and benefit from the emulsions’ high interfacial area, this research focuses on cellulose as a natural surfactant for oil-in-water emulsions. Cellulose is a biopolymer composed of D-glucose with β (1–4) links and is one of the most abundant biomasses in nature found in many plants, trees and algae [14]. It has a wide array of applications in the raw or processed food, cosmetics and pharmaceutical industries [15,16]. The amphiphilic nature of cellulose, exhibiting both hydrophilic and hydrophobic characteristics, facilitates its adsorption to both oil and water interfaces [17,18,19].

It can thus be utilized to form stable cellulose-coated oil-in-water emulsions [20,21,22,23]. Moreover, the particles exhibit a unique inner structure: the hydrophobic core is covered by a shell of aqueous cellulose hydrogel encased in an outer continuous shell of amorphous cellulose [22,23].

Previous studies have shown that enzymes can be combined with cellulose-coated emulsion microparticles. Cellulolytic enzymes can be attached to the outer coating surface for effective cellulose hydrolysis [24]. The integration of yeasts (*S. crevisiae*) with emulsion particles combined with cellulases provided effective emulsion-based simultaneous saccharification and fermentation (e-SSF) [25]. This is the basis of a ‘one-pot’ methodology for the conversion of cellulose to biofuel [26] and chemicals by consolidated bioprocess (CBP) using integrated enzymes and yeasts with cellulose-coated emulsion microparticles.

This study aims at exploring the ability of lipase incorporated in cellulose-coated emulsion particles to catalyze the transesterification of long-chain esters dissolved in the emulsion droplets’ core. The specific objectives are: to fabricate and characterize the structure of emulsion particles suitable for this purpose, to show that lipase added to the aqueous emulsion medium performs their catalytic function within the particles and not in the external medium and to investigate the catalytic activity of the incorporated lipase towards the transesterification of long-chain esters dissolved in the emulsion droplets’ core. Using model long-chain ester compounds, this study provides the first proof-of-concept that lipase introduced in the aqueous phase of the emulsion is incorporated with the emulsified particles and can catalyze transesterification rather than hydrolysis in such an aqueous system (more than 90% wt. water), where both reactants are located in the hydrophobic phase of cellulose-coated oil-in-water emulsion particles.

## 2. Results

The objective of this study was to utilize cellulose-coated oil-in-water emulsion particles as microreactors for lipase-catalyzed reactions. Thus, the particles’ structure was characterized in relation to its effect on the activity of lipase, incorporated into the microparticles by addition to the emulsion’s aqueous medium. Lipase activity was evaluated first in the hydrolysis of a fatty acid ester within the particle’s hydrophobic core. The ultimate goal of the study was to evaluate the activity of the incorporated lipase on promoting the transesterification reaction between fatty acid esters within the hydrophobic core of the particles, in competition with hydrolysis due to the overwhelming aqueous medium. The particles were fabricated from the cellulose hydrogel concentration of 1% wt., mixed with the increasing content of the hydrophobic phase. n-Decane was used as a model hydrophobic phase, being a good solvent for the tested substrates while enabling high lipase stability due to its high-ranking log *p*-value of 5.6 [27,28] (a parameter ranking of organic solvents by polarity for biocatalysis) [29] and a moderate viscosity suited for the emulsion fabrication process. The emulsion microparticles’ structure was investigated using light microscopy, fluorescence microscopy and cryo-SEM.

**Figure 1:**
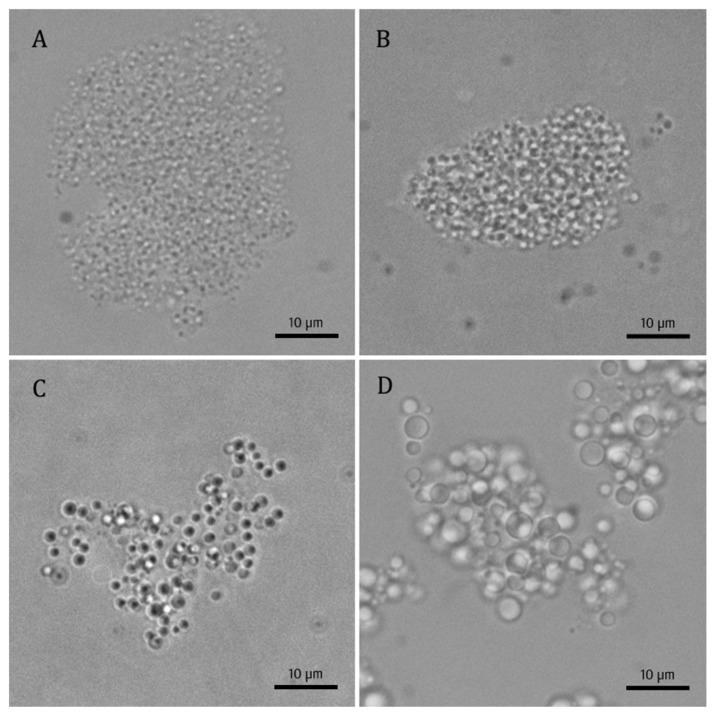
Light microscopy images (phase contrast) of cellulose-coated n-decane emulsion microparticles at cellulose:decane wt. ratios of: (**A**) 1:1, (**B**) 1:3, (**C**) 1:5 and (**D**) 1:8.

**Figure 2:**
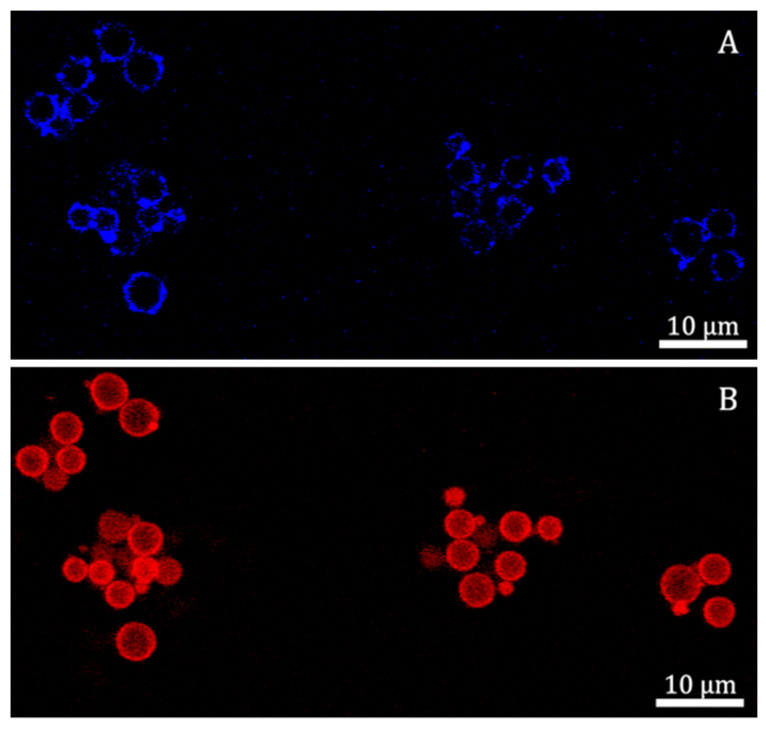
Fluorescence microscopy images of a 1:8 wt. ratio cellulose:decane emulsion stained with: (**A**) calcofluor white, preferentially staining cellulose, and (**B**) Nile red, preferentially staining the decane core.

Light microscope images of the emulsions, shown in Figure 1, exhibit cellulose-coated oil-in-water emulsion droplets having circular shapes with clear visible borders. The particles seem to appear as aggregates, with some isolated particles. A positive correlation can be found between the particle size and n-decane content (Figure 1), as consistent with previous studies [24]. The light scattering analysis (Figure S2, Supplemental Materials) exhibited a bimodal distribution of particle dimensions, confirming the coexistence of isolated particles, a few micrometers in dimension, with aggregates about 10 μm in size, as well as the increased particle dimension with decane content. The differentiation of cellulose in the encapsulation shell from the hydrophobic droplets’ core was confirmed using fluorescence microscopy, as shown in Figure 2, for emulsions of the cellulose:decane wt. ratio 1:8. The particles fabricated at this cellulose:decane ratio were chosen for visualization, since their size is most suitable for differentiating between the core and shell by fluorescence microscopy and visualization of the inner shell’s structure by SEM. The cellulose shell is imaged using calcofluor white (Figure 2A), whereas the hydrophobic core is imaged using Nile red (Figure 2B).

**Figure 3:**
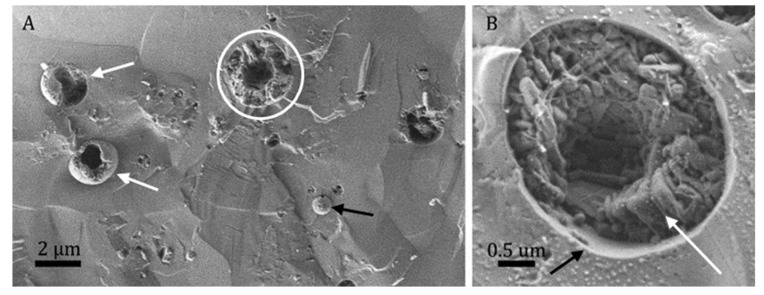
Cryo-SEM images of 1:8 (wt. ratio) cellulose:decane emulsion after cryo-fracturing. (**A**) Lower magnification exhibiting a few microparticles: whole (black arrow), partially fractured (white arrow) and fully fractured (white circle). (**B**) The two-shell encapsulation structure revealed at higher magnification imaging of the fully fractured emulsion particle: a thick porous inner shell (white arrow) with a thin and more compact outer shell (black arrow).

The inner structure and content of cellulose-coated emulsion particles at a wt. ratio of 1:8 cellulose:decane was investigated using cryo-SEM (Figure 3). The image shown in Figure 3A exhibits particles in vitrified emulsion at various states revealed by the cryo-fracturing and subsequent sublimation steps of sample preparation prior to imaging: whole and intact (black arrow), partially fractured (white arrow) and fully fractured (white circle). The latter two appear empty, probably due to removal of the exposed core during cryo-fracturing. A higher magnification image of the fully fractured emulsion particle, Figure 3B, reveals a double-shell structure composed of a thick porous inner shell (white arrow) with a thin and more compact outer shell (black arrow). It is consistent with the structure described previously for emulsions prepared from cellulose solution in ionic liquid, interpreting the inner shell to be composed of a cellulose hydrogel [20]. It was further verified recently in emulsions fabricated by a method similar to the one used in this study [23]. The thickness and porosity characteristics of the encapsulation shell structure are expected to be major factors in lipase’s ability to penetrate the droplet and reach the inner oil core interface to perform the desired catalytic function.

**Scheme 1:**
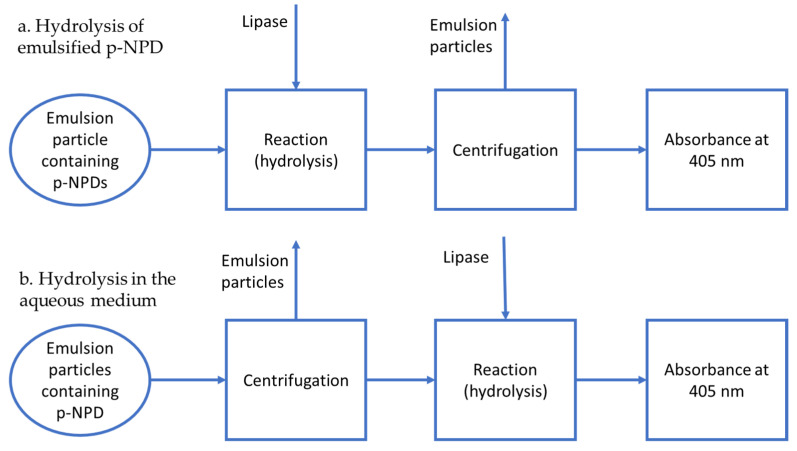
Experimental scheme designed to evaluate lipase activity in the emulsion’s inner core interface. Lipase-catalyzed hydrolysis: (**a**) in the emulsion and (**b**) in the aqueous medium.

The ability of the lipase, introduced into the aqueous emulsion medium, to pass through the cellulose shell of the emulsion particle and to catalyze a reaction in the inner core interface was evaluated using *p*-nitrophenyl dodecanoate (p-NPD) as a substrate. For this purpose, the p-NPD substrate was dissolved in the n-decane core before emulsification. The experimental steps evaluating the lipase-catalyzed hydrolysis of encapsulated p-NPD are described in Scheme 1a. The control experiment to negate lipase activity on non-emulsified p-NPD is described in Scheme 1b, in which the emulsion particles were centrifuged out before addition of the enzymes (at the same quantity).

**Figure 4:**
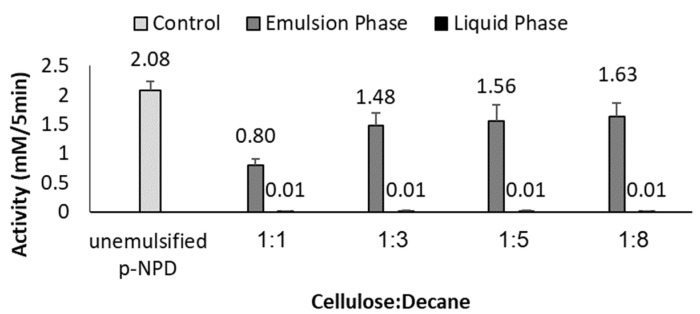
Lipase hydrolysis activity in 1:1, 1:3, 1:5 and 1:8 cellulose:decane emulsions. Comparison of lipase addition before and after centrifugation (Scheme 1a,b, respectively) and to lipase activity in an aqueous medium (control).

The hydrolysis of p-NPD in cellulose-coated microparticle emulsion was evaluated for different cellulose:decane ratios (1:1, 1:3, 1:5 and 1:8 wt.). The results showed that lipase can catalyze a hydrolysis reaction with activity of 38–76% compared to that of the control sample (Figure 4). Furthermore, only negligible activity of free lipase was found in the water (less than 1%), indicating that p-NPD was fully encapsulated in the core of the emulsion. This strengthened the suggested mechanism in which lipase reaches the internal particle core interface through the cellulose shell to effectively catalyze the hydrolysis reaction. The results also revealed that hydrolysis depends on the dimensions of emulsion particles. Higher hydrolysis rates were found in larger particles with high oil contents and, hence, a thinner shell. The lower hydrolysis of p-NPD in 1:1 cellulose:decane wt. ratio emulsion particles may be due to the relatively thicker shell due to the significantly higher cellulose content.

**Figure 5:**
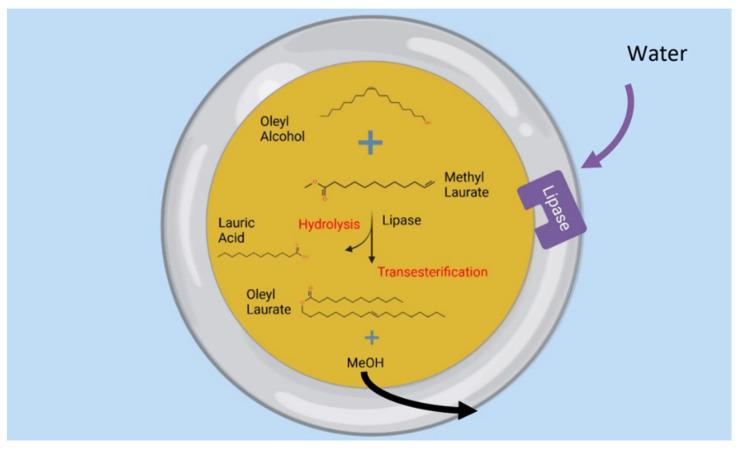
Illustration of the reaction sequence for lipase transesterification. Dark yellow—oil core. Gray cellulose outer shell. Light blue—water phase.

**Figure 6:**
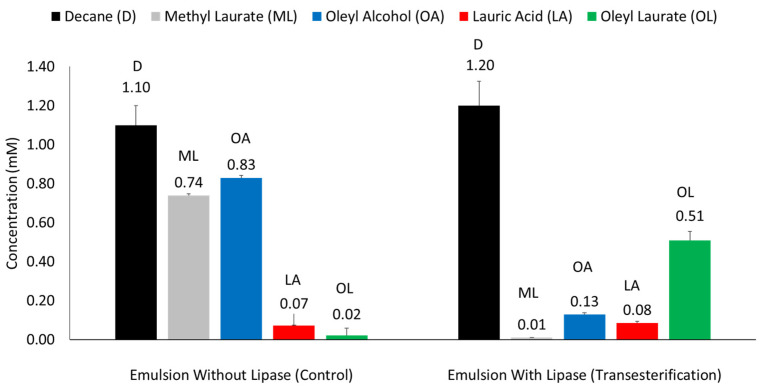
Concentrations of products from a lipase-catalyzed reaction in emulsion droplets comprising methyl laurate and oleyl alcohol. An analysis was performed using GC-MS. Bars represent the average results of the concentration, whereas the error bars represent the standard deviation.

Based on the findings that lipase introduced into the aqueous medium of the cellulose-coated microparticle emulsion can effectively hydrolyze fatty acid ester dissolved in the particles’ hydrophobic core, the activity of the incorporated lipase in a transesterification reaction between fatty acid esters rather than hydrolysis was assessed. For this purpose, emulsion particles were fabricated with two long-chain compounds as reactants (methyl laurate-ML and oleyl alcohol-OA) dissolved in the hydrophobic core. These substrates were mixed with decane at concentrations of 1.1 mM and 0.93 mM, respectively, and fabricated to an emulsion using a 1:1 wt. ratio of cellulose to the hydrophobic phase. This ratio was chosen in view of ongoing research on a CBP described in the introduction section. It is based on the integration of cellulose-coated emulsion particles with yeasts to provide a cascade of biochemical reactions: cellulose hydrolysis [24], glucose fermentation [25] and lipase-catalyzed transesterification of triglycerides [30]. The utilization of cellulose as the substrate for alcohol (glucose and ethanol) in the integrated system requires a thicker coating. It is therefore interesting to evaluate whether lipase can penetrate a thicker shell to function catalytically at the inner core–shell interface, as both reactants are soluble in the core. The full reaction scheme is detailed in Figure 5. In this experiment, lipase was introduced into the aqueous phase, the reaction products were analyzed using GC-MS and the results are presented in Figure 6, together with a control experiment without the lipase, to negate the effect of a spontaneous reaction.

## 3. Discussion

The reported results revealed that lipase can catalyze transesterification and produce oleyl laurate in cellulose-coated oil-in-water emulsion when both reactants are located in the hydrophobic phase. In this case, transesterification is driven by the release of methanol into the aqueous emulsion medium. Only negligible hydrolysis occurred, as only a minute amount of lauric acid was observed. To further prove that transesterification occurred due to the emulsion structure, a reaction using the same reactants dissolved in decane without emulsification was conducted. This reaction did not produce any noticeable amounts of ester. The novelty of this result lies in the ability of lipase to catalyze transesterification rather than the anticipated hydrolysis when the reaction is performed in an aqueous environment with more than 90% wt. water and both reactants are located in the hydrophobic phase. This result exhibits an important development in the field of biocatalysis in organic solvents, since it is well-known that the presence of water preserves the lipase structure and activity [31,32], whereas too much water shifts the reaction to hydrolysis rather than transesterification, and increasing the water content even above 12% wt. affects the transesterification [33,34]. Therefore, this proves that lipase reaches the emulsion particles, penetrates the cellulose hydrogel core and catalyzes a transesterification reaction in the hydrophobic phase. This may be beneficial compared to a reaction in a nonaqueous solvent environment, as it may require smaller solvent quantities.

Micro-structured particles for the confinement of biocatalysts in compartmentalized microenvironments are considered the key step towards biomimetic microreactors. For example, the design of a three-tiered structured microparticle (“colloidosome”) by Pickering emulsion for lipase-catalyzed hydrolysis of a long-chain ester was recently reported. It consists of crosslinked amphiphilic silica-polymer nanoparticles that form an outer shell with an enzyme-incorporated aqueous catalytic sublayer surrounding the inner microparticle core [35]. This water-in-oil emulsion is the reverse of the one reported in the current study, but it shares the relevance of a multi-shelled emulsion particle structure for enhanced biosynthesis. The importance of a suitable “micro-water environment” for lipase activity is highlighted in a recent report on the esterification of ethanol with conjugated linoleic acid by lipase incorporated in polymer hydrogel microparticles [36]. Whereas, in these examples, the hydrophobic phase is the continuous medium, a lipase-catalyzed reaction was reported in an oil-in-water (O/W) Pickering emulsion by self-assembly of lipase/chitosan nanoparticles, which function as both stabilizer and catalyst, in emulsion droplets as microreactors with reactants in the oil core [37].

The production of oleyl laurate, a large molecule compared to common fatty acid ethyl or methyl esters, in the emulsion particles exhibits a potential for the presented cellulose emulsion system to serve as a microbioreactor for the transesterification of triglycerides and other high molecular weight esters in an aqueous medium. It may be expected that emulsion-based biocatalytic processes can operate under mild aqueous reaction conditions and utilize nontoxic reagents with biodegradable effluents without compromising the conversion and selectivity [38,39]. In particular, erythorbyl myristate (EM), a potential multifunctional food emulsifier, was newly synthesized by immobilized lipase-catalyzed esterification between antioxidative erythorbic acid and antibacterial myristic acid [40]. The significance of the reported observations is related to the transformation of biomass-based feedstocks into valorized chemicals by biocatalysis. For example, the incorporation of cellulolytic enzymes with the cellulose-coated emulsion microparticles containing lipase in the inner cellulose hydrogel shell may be utilized for a cascade of consolidated bioprocesses for the synthesis of sugar-based amphiphiles.

## 4. Materials and Methods

### 4.1. Materials

The enzyme *Thermomyces lanuginosus* lipase and microcrystalline cellulose powder (Avicel^®^) were purchased from Sigma Aldrich (Rehovot, Israel). The chemicals p-nitrophenyl dodecanoate (p-NPD), 4-nitrophenol (4-NP), acetonitrile, Nile red, calcofluor white (CFW), lauric acid (LA) pharmaceutical standard and methyl laurate (ML) >98% food grade were also purchased from Sigma Aldrich. Sodium hydroxide and isopropanol were purchased from Bio-Labs (Jerusalem, Israel), n-decane from Mercury (Karlsfeld, Germany), hexane from Frutarom (Haifa, Israel), the Tris base from Spectrum (New Brunswick, NJ, USA), oleyl alcohol (OA) technical grade from Thermo Fisher Scientific (Braunschweig, Germany) and oleyl laurate GC grade (OL) from Larodan (Solna, Sweden). All materials were used as received.

### 4.2. Emulsion Fabrication and Characterization

NaOH was dissolved in deionized water (7% wt.) at room temperature. Microcrystalline cellulose was mixed with sodium hydroxide solution (7% wt.). Dissolution was achieved by cooling the sample to −20 °C while continuously stirring (500 rpm). The solution was then diluted with deionized water until the electrical conductivity (CD-2014YK, Lutron, Ramat Gan, Israel) was <1 mS/cm at ambient temperature, resulting in the formation of regenerated cellulose hydrogel. Excess water was removed using Whatman glass fiber filter paper, and the cellulose concentration of the formed hydrogel suspension was determined gravimetrically. The cellulose emulsion was fabricated by mixing 10 mg/mL cellulose hydrogel suspension with n-decane (the hydrophobic phase). The hydrogel (determined to contain 1% wt. cellulose) was mixed with the hydrophobic phase and homogenized mechanically (T-18 Ultra-Turrax, IKA Works, Staufen, Germany) for 25,000 rpm (10 min) to reduce the particle size and then emulsified using a high-pressure homogenizer (LM-20, Microfluidics, Newton, MA, USA) at 10 kPSI (4 min) while maintaining a sample temperature below 40 °C.

Analysis of the emulsion droplets’ size was performed using a light microscope equipped with achromatic positive low-phase contrast objectives (Olympus, Tokyo, Japan). Images were taken with a 12-bit cooled CCD camera (Sensicam PCO, Kelheim, Germany) and measurements performed using image analysis software (ImageJ, National Institute of Health, Bethesda, MD, USA). Emulsion particle size distribution was measured in triplicate by light scattering (DLS) (Mastersizer 2000 Malvern Co., Malvern, UK). Imaging of the emulsion droplets’ structure was performed by cryogenic scanning electron microscopy (cryo-SEM, Carl Zeiss, Oberkochen, Germany) using a Zeiss Ultra Plus microscope equipped with a Schottky field-emission gun. A Bal-Tec VCT100 (Leica Microsystems, Wetzlar, Germany) cold stage maintained at a temperature below −145 °C was used to hold the samples. Imaging was performed at a low electron acceleration voltage (1–1.4 kV) to avoid charging without conductive coating of the imaged surface and at a working distance of 3.2–3.8 mm using an Everhart–Thornley secondary electrons detector (SE2, Carl Zeiss, Oberkochen, Germany). Specimens for cryo-imaging were prepared by placing an emulsion droplet on a stub that was vitrified by plunging into supercooled liquid ethane followed by liquid nitrogen. Frozen samples were transferred to a freeze fracture unit (BAF060) via a pumped cryo-transfer shuttle maintained at −170 °C. Frozen samples were then fractured by a rapidly cooled knife and transferred to imaging at the SEM. Fluorescence microscope images were taken using a laser scanning confocal microscope (LSM700, Zeiss, Oberkochen, Germany) equipped with a Meta detector. Samples were stained with calcofluor white (CFW) and Nile red, which stain the cellulose and oil, respectively. CFW is fluorescent in green or blue color by excitation at 350 nm and emission at 450–500 nm, and Nile red, a hydrophobic dye, has fluorescence with an excitation at 515–560 nm and emission at 590 nm [20].

### 4.3. Lipase Activity Evaluation

Lipase-catalyzed hydrolysis was evaluated by colorimetric measurement of the aqueous medium (at 405 nm, using a pre-calibrated absorption curve) due to 4-NP released by the hydrolysis of p-NPD dissolved in the emulsion droplets’ hydrophobic core at the final concentration of 5 mM. Since light scattering from the emulsion particles interferes with the spectroscopic absorbance measurement at 405 nm due to 4-NP released by p-NPD hydrolysis, the emulsion particles were centrifuged out 5 min after the enzymes were introduced to the aqueous phase at a concentration of 0.7 ng/mL. The sequence of steps in the hydrolysis and control experiments is described schematically in Scheme 1 in the Results section. In both experiments, the reaction time was 5 min at room temperature. Colorimetric measurements were performed after incubation with buffer solution for 5 min at ambient temperature. The reaction buffer was prepared by mixing 95% vol., 50 mM Tris-HCl, 4% vol. isopropanol and 1% vol. acetonitrile. As a further control, the lipase activity was compared with the hydrolysis of p-NPD dissolved in isopropanol in the reaction buffer without emulsion particles. The activity was evaluated as the amount of 4-NP created after a 5-min reaction. All hydrolysis experiments were performed in triplicate.

The ability of lipase to initiate and accomplish the transesterification of substrates within the hydrophobic core of the emulsion droplets, in competition with hydrolysis by the omnipresent water, was evaluated by introducing lipase in the emulsion system containing ML (ester) and OA (alcohol) located in the hydrophobic emulsion droplets’ core. Emulsions were fabricated at the cellulose:oil ratio of 1:1 (wt.), containing ML and OA at concentrations of 1.1 mM and 0.93 mM, respectively. Then, 7 µg/mL lipase (in excess) was added to a 50 mL emulsion sample and incubated in a radial shaker for 24 h at 40 °C at 200 rpm. After incubation, the samples were frozen overnight and defrosted to break the emulsion droplets and centrifuged at 1200× *g* for 5 min. The supernatant (oil phase) was removed for analysis by gas chromatography (GC). Positive control samples contained DI water instead of cellulose hydrogel, and the negative control contained all the ingredients but without lipase. The contents of decane, ML, LA, OA and OL were quantified using a 6890 N GC instrument (Agilent Technologies, CA, USA) equipped with a capillary HP-5MS column (30 m × 250 μm × 0.25 μm, Agilent Technologies) and 5975 Mass Selective Detector (MSD) System (Agilent Technologies, CA, USA). Samples (1 µL) were injected in split mode (1/10). The initial column temperature was 40 °C and kept for 1 min, raised to 240 °C at 50 °C/min and raised to 320 °C at 10 °C/min, then maintained at this temperature for 1 min. Helium was used as a carrier gas at a column flow rate of 1 mL/min [41]. Under this program’s conditions, the retention times of decane, ML, LA, OA and OL were 2.60, 4.33, 4.43, 5.78 and 11.18 min, respectively (See Figure S1 in the Supplemental Materials).

## 5. Conclusions

This article demonstrates lipase activity in cellulose-coated emulsion microparticles with a unique structure in which the enzyme functions at the interface between the hydrophobic core and an inner shell composed of cellulose hydrogel. This provides a suitable microenvironment for lipase activity. It was shown that lipase thus incorporated can effectively catalyze the transesterification of long-chain molecules (methyl laurate and oleyl alcohol) dissolved in the core solvent (decane). In this system, lipase successfully catalyzed the transesterification reaction rather than hydrolysis, despite the dominant presence of water in the emulsion medium.

This research exhibits a potential for the cellulose emulsion system to serve as a microbioreactor for the transesterification of triglycerides and other high molecular weight esters in an aqueous medium. It can be used as a replacement for expensive catalysts and ease the product separation process. Furthermore, it can be used for consolidated bioprocesses based on the integration of cellulose-coated emulsion particles with yeasts to provide a cascade of biochemical reactions.

## Data Availability

Not applicable.

## Supplementary Materials

The following supporting information can be downloaded at https://www.mdpi.com/article/10.3390/ijms232012122/s1.
