# Supplementary material for: Lipase Catalyzed Transesterification of Model Long-Chain Molecules in Double-Shell Cellulose-Coated Oil-in-Water Emulsion Particles as Microbioreactors
Source: Int J Mol Sci. 2022 Oct 12;23(20):12122. doi: 10.3390/ijms232012122 (PMC9603428; doi:10.3390/ijms232012122)
Supplement: Supplementary file 1 [file ijms-23-12122-s001.zip › ijms-1937600-supplementary.pdf]

International Journal of *Molecular Sciences*

Article

Lipase Catalyzed Transesterification of Model Long-Chain Molecules in Double-Shell Cellulose-Coated Oil-in-Water Emulsion Particles as Microbioreactors

Itzhak Meir [1,†], Gilad Alfassi [2,†], Yael Arazi [3], Dmitry M. Rein [1], Ayelet Fishman [3] and Yachin Cohen [1,*]

[1] Department of Chemical Engineering, Technion—Israel Institute of Technology, Haifa 3200003, Israel
[2] Department of Biotechnology Engineering, Braude College of Engineering, Karmiel 2161002, Israel
[3] Department of Biotechnology and Food Engineering, Technion—Israel Institute of Technology, Haifa 3200003, Israel
* Correspondence: yachinc@technion.ac.il
† These authors contributed equally to this work.

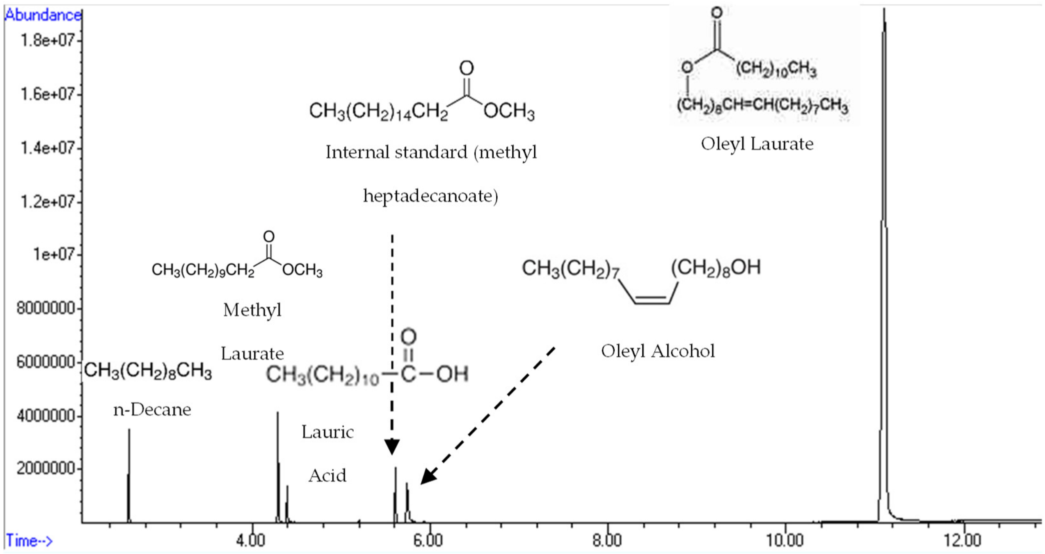

Figure S1. GC-MS chromatogram of a mixture containing (from left to right): decane, metyl laureate, lauric acid, internal standard (methyl heptadecanoate), oleyl alcohol and oleyl laureate.

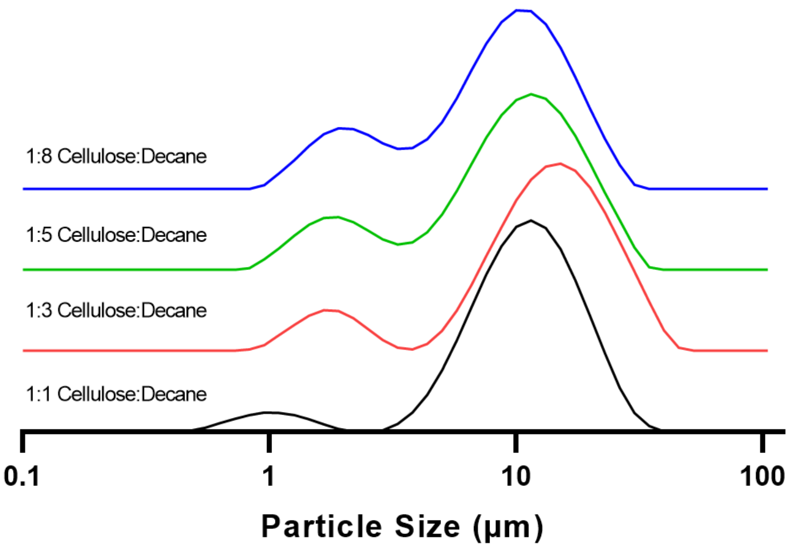

Figure S2. Particle size distribution of emulsified n-decane with cellulose hydrogel at 1:1, 1:3, 1:5, 1:8 cellulose:oil ration, determined by light scattering.
